# Tomato LysM receptor kinase 4 mediates chitin-elicited fungal resistance in both leaves and fruit

**DOI:** 10.1093/hr/uhad082

**Published:** 2023-04-25

**Authors:** Yingfei Ai, Qinghong Li, Chenying Li, Ran Wang, Xun Sun, Songyu Chen, Xin-Zhong Cai, Xingjiang Qi, Yan Liang

**Affiliations:** State Key Laboratory for Managing Biotic and Chemical Threats to the Quality and Safety of Agro-products, Department of Plant Protection, Zhejiang University, Hangzhou, 310058, China; State Key Laboratory for Managing Biotic and Chemical Threats to the Quality and Safety of Agro-products, Department of Plant Protection, Zhejiang University, Hangzhou, 310058, China; State Key Laboratory for Managing Biotic and Chemical Threats to the Quality and Safety of Agro-products, Department of Plant Protection, Zhejiang University, Hangzhou, 310058, China; State Key Laboratory for Managing Biotic and Chemical Threats to the Quality and Safety of Agro-products, Department of Plant Protection, Zhejiang University, Hangzhou, 310058, China; State Key Laboratory for Managing Biotic and Chemical Threats to the Quality and Safety of Agro-products, Department of Plant Protection, Zhejiang University, Hangzhou, 310058, China; State Key Laboratory for Managing Biotic and Chemical Threats to the Quality and Safety of Agro-products, Department of Plant Protection, Zhejiang University, Hangzhou, 310058, China; Hainan Institute, Zhejiang University, Sanya, 572025， China; Zhejiang Xianghu Laboratory, Institute of Horticulture, Zhejiang Academy of Agricultural Sciences, Hangzhou, 310021, China; State Key Laboratory for Managing Biotic and Chemical Threats to the Quality and Safety of Agro-products, Department of Plant Protection, Zhejiang University, Hangzhou, 310058, China

## Abstract

Fungal infection is a major cause of crop and fruit losses. Recognition of chitin, a component of fungal cell walls, endows plants with enhanced fungal resistance. Here, we found that mutation of tomato LysM receptor kinase 4 (SlLYK4) and chitin elicitor receptor kinase 1 (SlCERK1) impaired chitin-induced immune responses in tomato leaves. Compared with the wild type, *sllyk4* and *slcerk1* mutant leaves were more susceptible to *Botrytis cinerea* (gray mold). SlLYK4 extracellular domain showed strong binding affinity to chitin, and the binding of SlLYK4 induced SlLYK4-SlCERK1 association. Remarkably, qRT–PCR analysis indicated that *SlLYK4* was highly expressed in tomato fruit, and *β-GLUCURONIDASE* (*GUS*) expression driven by the *SlLYK4* promoter was observed in tomato fruit. Furthermore, *SlLYK4* overexpression enhanced disease resistance not only in leaves but also in fruit. Our study suggests that chitin-mediated immunity plays a role in fruit, providing a possible way to reduce fungal infection-related fruit losses by enhancing the chitin-induced immune responses.

## Introduction

In nature, plants constantly face a wide range of pathogens, especially fungal pathogens, which cause severe yield loss of crops. Through co-evolution with pathogens, plants have evolved an immune system to defend themselves against pathogen attacks [1]. The immune signaling is initiated by pattern recognition receptors (PRRs) at the plasma membrane, which often recognize the conserved molecular patterns from pathogens, namely pathogen-associated molecular patterns (PAMPs) [2, 3]. Activation of PRRs triggers basal plant defenses against a broad spectrum of microbial infections. For example, in several crops, ectopic expression of an *Arabidopsis* ELONGATION FACTOR-TU RECEPTOR (EFR), a PRR recognizing a conserved bacterial PAMP EF-Tu, rendered plants more resistant to a range of different bacteria [4, 5]. Therefore, manipulating PRR and PAMP signaling provides a potential way to engineer plants to achieve durable and sustainable resistance [6–9].

Chitin, the major component of the fungal cell wall, is one of the most widely known fungal PAMPs [10]. In addition, chitin is found in the exoskeleton of crustaceans and insects but not in mammals [11, 12]. Chemically, chitin is a polysaccharide made of *N*-acetyl-d-glucosamine (GlcNAc) and is the second most abundant polysaccharide after cellulose [13, 14]. Plant chitinases hydrolyze the polymer chitin to chitooligosaccharides following fungal infection [15, 16]. Degrees of polymerization between 6 and 8 (CO_6_–CO_8_) elicit plant immune responses, such as the production of reactive oxygen species (ROS), calcium influx, phosphorylation of mitogen-activated protein (MAP) kinase (MAPK), and callose depositions, resulting in the restriction of fungal infection [11, 12, 17, 18]. Generally, chitin perception activates a basal resistance of plants against a broad range of pathogenic fungi.

Chitin receptors belong to a family of receptor kinases containing the lysin motif (LysM), a conserved domain with ~40 amino acid repeats and first identified in the lysozyme of a bacteriophage [19, 20]. In rice (*Oryza sativa*), chitin is recognized by CHITIN ELICITOR-BINDING PROTEIN (OsCEBiP), a LysM receptor-like protein (LYP) located on the plasma membrane via a glycosylphosphatidylinositol (GPI) anchor [21, 22]. In addition, LYP4 and LYP6 were also reported to bind chitin and play a redundant role in the recognition of chitin in rice [23]. All these LYPs have no intracellular kinase domain but form heterodimers with CHITIN ELICITOR RECEPTOR KINASE1 (OsCERK1) after chitin recognition, thus transducing immune signals from the plasma membrane to the cytosol through the activation of the OsCERK1 intracellular kinase domain [24, 25]. Chimeric receptors consisting of OsCEBiP fused with the intracellular kinase domain of other PRRs (Xa21, a receptor mediating disease resistance to rice bacterial leaf blight) enhance chitin-triggered immunity and confer resistance to rice blast, the most serious fungal rice disease [26]. In addition, OsCERK1 was found to play roles in identifying peptidoglycan from bacteria, oligosaccharides containing β-1,3-1,4-glucan from plant cell walls, and even molecules from symbiotic arbuscular mycorrhiza [27–30]. In *Arabidopsis thaliana*, an ortholog of OsCEBiP, AtLYM2, does not seem to mediate CERK1-dependent chitin responses; however, it regulates chitin-induced plasmodesmata flux in a CERK1-independent manner [31]. As a replacement for OsCEBiP, chitin is recognized by other LysM receptor kinases (LYKs), AtLYK4 and AtLYK5 in *Arabidopsis*, which contain the kinase domain but have lost the kinase activity because of the lack of amino acids essential for kinase activity [32]. *Lyk4 lyk5* double mutants almost abrogated chitin-induced immune responses [32]. Although the binding receptors of chitin differ between rice and *Arabidopsis*, they transduce signals via an identical mechanism of complex formation with OsCERK1 [24, 32]. Phylogenetic analysis suggested that chitin receptors in dicots clustered with AtLYK4/5 in one clade, whereas those in monocots clustered with OsCEBiP in another clade, implying that LYK members may have evolved before the divergence of monocots and dicots [33]. To date, chitin receptors have been identified in many other plant species, such as *Lotus japonicus*, *Brassica juncea*, and cotton (*Gossypium hirsutum*), and it has been elucidated that chitin receptors mediate basal resistance to fungal infection in these plant species [34–36]. However, all these studies were done in leaves or roots; it is still unclear whether chitin-mediated disease resistance plays a role in fruit.

Fruits are highly susceptible to many fungal pathogens, among which *Botrytis cinerea*, which causes gray mold, is considered one of the most destructive pathogens for fresh fruits and vegetables [37, 38]. Tomato (*Solanum lycopersicum*) has become a model for studying disease resistance in fruit because of its economic and nutritional value. In addition to tomato, *B. cinerea* infects many other fresh fruits, such as strawberry, grape, blackberry, blueberry, raspberry, and several others. To reduce the use of chemical fungicides, several strategies have been investigated to control post-harvest decay of fruits and vegetables, including physical treatments, natural antimicrobials, and plant immunity stimulators [39–42]. Therefore, understanding chitin-induced immunity in fruit is a possible way of developing a cultivar with resistant fruit or identifying physical factors and immunity stimulators that could induce chitin immunity to defend against fungal infection.

In this study, we generated tomato chitin receptor mutants, *sllyk4* and *slcerk1*, using CRISPR/Cas9 technology and investigated the chitin-induced immunity and fungal resistance. We demonstrated a conserved model of chitin recognition in tomatoes. In addition, we found that *SlLYK4* was highly expressed in tomato fruit and induced by low temperature and CaCl_2_ addition. SlLYK4 mediated chitin-elicited fungal resistance in fruit, and overexpression of *SlLYK4* enhanced tomato fruit resistance to *B. cinerea* infection.

## Results

### *SlLYK4*-silencing plants show reduced chitin responses

**Figure 1:**
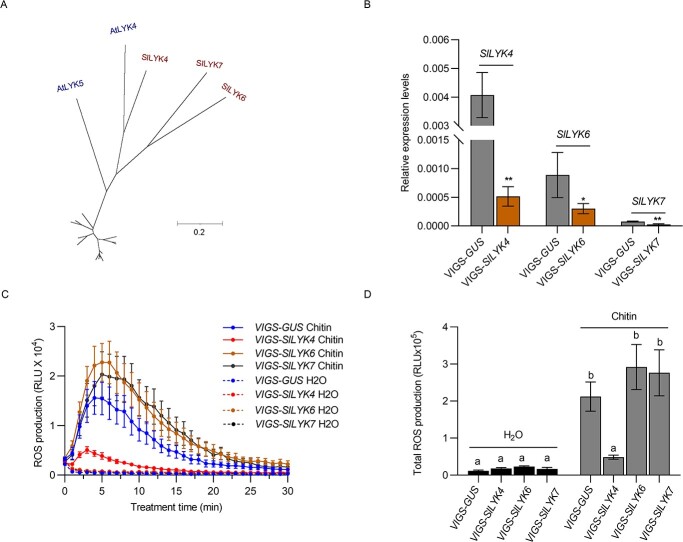
*SlLYK4*-silencing leaves show reduced chitin responses. (A) AtLYK5 subclade. SlLYK4, SlLYK6, and SlLYK7 are clustered with AtLYK4 and AtLYK5 into one subclade. A full phylogenetic tree with all LYKs from *Arabidopsis* and tomato is shown in Supplementary Data Fig. S1. Except for the AtLYK5 branch, all other branches are scaled down by a factor of 10. (B) Silencing efficiency of *VIGS*-*SlLYK4*, -*SlLYK6*, and -*SlLYK7*. The silencing of *SlLYK4*, *SlLYK6*, and *SlLYK7* was mediated by the VIGS approach. Silencing efficiency was compared between *VIGS-GUS* and its respective VIGS construct. The relative transcript levels were determined by qRT–PCR. *SlEF1α* was used as an internal control. Data are shown as mean ± standard deviation (*n* = 3–5). Asterisks indicate significant differences between the control and *VIGS* target (^*^P ≤ .05, ^**^P ≤ .01, *t*-test). (C, D) Production of ROS. ROS were measured using a chemiluminescence assay. ROS signals were recorded for 30 minutes after treatment with 50 μg/ml chitin (C), and total ROS production within 30 minutes is shown in (D). Data are expressed as mean ± standard error (*n* = 6–8). Different letters indicate significant differences between the control and *VIGS* target (*P* ≤ .05, one-way ANOVA).

Phylogenetic analysis using amino acid sequences of five LYKs from *Arabidopsis* and 14 LYKs from *S. lycopersicum* indicated that SlLYK4, SlLYK6, and SlLYK7 were clustered into one clade with AtLYK4 and AtLYK5 (Fig. 1A), whereas SlLYK1, SlLYK12, and SlLYK13 were in one group with AtCERK1 (Supplementary Data Fig. S1), which is consistent with previous phylogenetic analysis [43–45]. By silencing individual members of the AtCERK1 clade using a VIGS approach, we previously identified that SlLYK1 (SlCERK1 hereafter) was subfunctionalized for chitin signaling in tomato [46]. Likewise, we silenced individual tomato orthologs in the *AtLYK5* clade, including *SlLYK4*, *SlLYK6*, and *SlLYK7*, by VIGS (Fig. 1B). The transcript levels of each silenced gene were determined by qRT–PCR in the leaves of cognate VIGS plants compared with negative control plants that were infiltrated with *VIGS*-*GUS* construct (Fig. 1B). After the reduced transcript levels were confirmed, chitin-induced ROS production was measured by a chemiluminescent assay in those leaves. Compared with the control, *SlLYK4*-silenced plants showed significantly reduced ROS levels after chitin treatment, whereas *SlLYK6*- and *SlLYK7*-silenced plants did not (Fig. 1C and D). Furthermore, the transcripts of *SlLYK4* were induced in leaves and roots after chitin treatment, but *SlLYK6* and *SlLYK7* were not (Supplementary Data Fig. S2). These results suggest that SlLYK4 might be the primary member responsible for chitin perception in tomatoes.

### *sllyk4* and *slcerk1* mutants show reduced chitin responses in leaves

**Figure 2:**
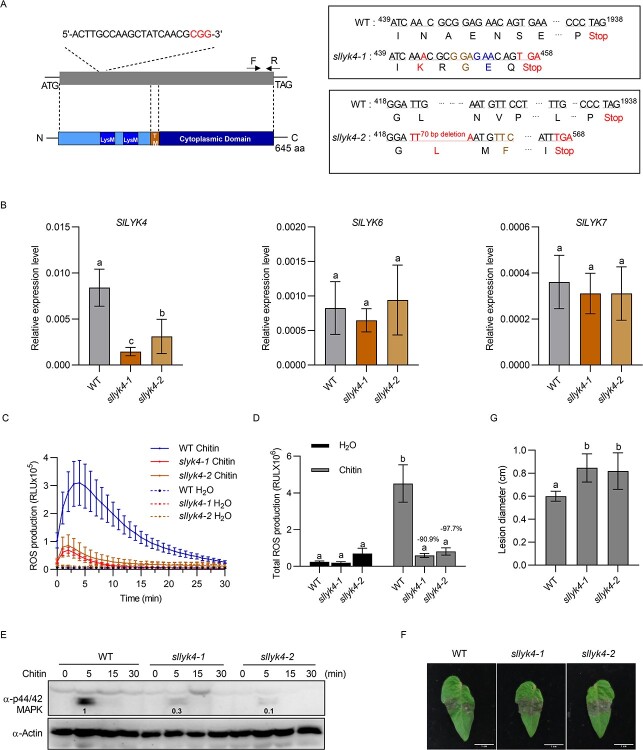
Leaves of *sllyk4* mutant show reduced chitin responses. (A) Schematic representation of *sllyk4* mutants. The *sllyk4* mutants were generated by CRISPR/Cas9 technology. The gray box indicates the exon of *SlLYK4*; the gRNA sequences for CRISPR/Cas9 are shown above it and the structural features of SlLYK4 are shown below it. TM, transmembrane region (270–298 amino acids). The *sllyk4-1* line has a 1-bp insertion and the *sllyk4-2* line has a 70-bp deletion, resulting in premature stop codons at amino acids 152 and 189, respectively. (B) Relative transcript levels. RNA was extracted from 2-week-old wild type (WT) and *sllyk4* mutant leaves and roots. Transcript levels of *SlLYK4*, *SlLYK6*, and *SlLYK7* were determined by qRT–PCR. *SlEF1α* was used as an internal control. Data are expressed as mean ± standard deviation (*n* = 4). Different letters indicate significant differences between the WT and *sllyk4* mutants (*P* ≤ .05, one-way ANOVA). (C, D) Production of ROS. ROS levels were monitored using a chemiluminescence assay after treatment with 50 μg/ml chitin. The line graph for 30 minutes is shown in (C), and total ROS production is shown in (D). Data are presented as mean ± standard error (*n* = 6–8). Different letters indicate significant differences between WT and *sllyk4* mutants (*P* ≤ .05, one-way ANOVA). (E) Abundance of phosphorylated MAPK. Proteins were extracted from 8-day-old tomato cotyledons at the indicated time points after treatment with 50 μg/ml chitin. MAPK phosphorylation was detected via immunoblotting using an anti-p42/44 MAPK antibody. Actin served as a loading control. Band intensity was measured by ImageJ. Numbers on the blot indicate the relative levels of phosphorylated MAPK proteins in mutants normalized to those in WT. (F, G) *Botrytis cinerea* disease assay. Tomato leaves (6 weeks old) were detached and spot-inoculated with 2.5 μl of *B. cinerea* spores (1 × 10^5^ spores/ml). Images were taken 3 dpi. Representative images are shown in (F), and lesion diameters are shown in (G). Data are presented as mean ± standard deviation (*n* = 10). Different letters indicate significant differences between the WT and *sllyk4* mutants (*P* ≤ .05, one-way ANOVA).

To further elucidate the contributions of SlLYK4 and SlCERK1 to chitin-induced immunity, we generated stable transgenic mutants using CRISPR/Cas9 technology. gRNA was designed at the 5′ end of *SlLYK4* and 420 nt from the start codon, and two independent lines were isolated, *sllyk4-1* and *sllyk4-2*. The *sllyk4-1* mutant contains a 1-bp insertion at 444 nt in the *SlLYK4* gene, resulting in a frameshift and premature stop codon at amino acid 152 (Fig. 2A). The *sllyk4-2* mutant was characterized by a 70-bp deletion in the *SlLYK4* gene and a premature stop codon at amino acid 189 (Fig. 2A). Transcript levels of *SlLYK4*, *SlLYK6*, and *SlLYK7* were determined in two *sllyk4* mutants by qRT–PCR analysis. Reduced *SlLYK4* transcripts were found in two *sllyk4* mutants, whereas transcript levels of *SlLYK6* and *SlLYK7* had no significant difference from those in the wild type (Fig. 2B). Chitin-induced ROS production was significantly lower in *sllyk4-1* and *sllyk4-2* mutants than in the wild type (Fig. 2C and D). Phosphorylation of MAPK was detected via immunoblotting using an anti-p44/42 MAPK antibody. Compared with the wild type, *sllyk4-1* and *sllyk4-2* mutants showed reduced MAPK phosphorylation levels after chitin treatment (Fig. 2E). Furthermore, chitin-induced expression of defense-responsive genes, *SlWRKY33* and *SlWRKY53*, were also reduced in *sllyk4-1* and *sllyk4-2* mutants compared with the wild type (Supplementary Data Fig. S3). These results suggest that *sllyk4* mutants show reduced immune responses after chitin treatment.

**Figure 3:**
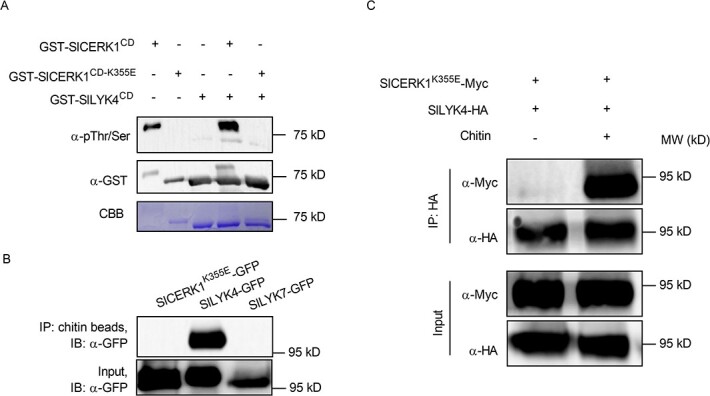
Recognition of chitin by SlLYK4 induces SlLYK4-SlCERK1 association. (A) SlLYK4 is kinase-inactive. The cytosolic kinase domains of the wild-type SlCERK1 (SlCERK1^CD^), kinase-dead version of SlCERK1^CD^ (SlCERK1^CD-K355E^) and SlLYK4^CD^ were N-terminally fused to a GST tag. Purified recombinant proteins were subjected to an *in vitro* kinase assay. Phosphorylated and input proteins were detected via immunoblotting using anti-pThr/Ser (Top) and anti-GST antibodies (middle), respectively. Coomassie brilliant blue (CBB) staining of the membrane is shown at the bottom. (B) SlLYK4 has a strong chitin-binding affinity. SlLYK4, SlLYK7, and a kinase-dead version of SlCERK1 (SlCERK1^K355E^) were fused to GFP and transiently expressed in *N. benthamiana*. Proteins were pulled down using chitin-magnetic beads. Chitin-binding and input proteins were detected via immunoblotting using an anti-GFP antibody. (C) Chitin induces SlCERK1-SlLYK4 association. HA-tagged SlLYK4 and Myc-tagged SlCERK1^K355E^ were co-expressed in *N. benthamiana*. Leaves were infiltrated with chitin (100 μg/mL) or ddH_2_O 2 days after co-expression and harvested 15 minutes after chitin treatment for protein extraction. Immunoprecipitation was carried out with anti-HA magnetic beads. Input and co-immunoprecipitated proteins were detected via immunoblotting with anti-HA and anti-Myc antibodies.

To investigate the potential contribution of *SlLYK4* to resistance to disease caused by the tomato fungal pathogen *B. cinerea*, we examined the disease phenotype of the *sllyk4* plants after inoculation with *B. cinerea* using a detached leaf inoculation assay (Fig. 2F and G). Under our disease assay conditions, necrotic lesions were observed in the leaves 3 dpi (Fig. 2F). The lesions in the *sllyk4* leaves expanded much more rapidly than in the wild type, and the lesion size in *sllyk4* leaves (average of ~8.5 mm) was 40% larger than that in the wild-type plants (average of 6.0 mm; Fig. 2F and G). The biomass of fungi detected by DNA-based qPCR in *sllyk4* mutants was significantly higher than in the wild type (Supplementary Data Fig. S4). In addition, we found that *sllyk4* mutants were more susceptible to *S. sclerotiorum* (Supplementary Data Fig. S5), a devastating soil-borne fungal pathogen causing tomato sclerotinia stem rot [47]. Collectively, these results suggest that *sllyk4* mutants show reduced immune responses after chitin treatment.

**Figure 4:**
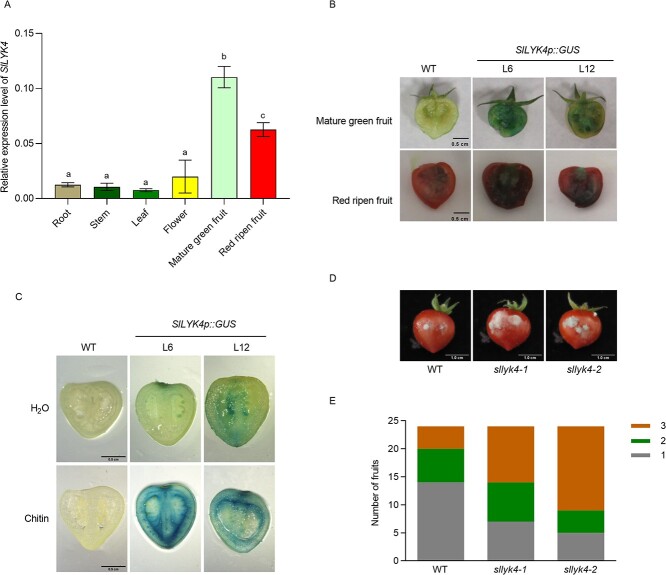
*SlLYK4* is highly expressed in fruit and mediates fruit resistance to *B. cinerea.* (A) Gene expression of *SlLYK4* in different tissues. RNA was extracted from root, stem, leaf, flower, and fruit of ‘Micro-Tom’. Transcript levels of *SlLYK4* were determined using qRT–PCR. *SlEF1α* served as an internal control. Data are presented as mean ± standard deviation of three independent replicates. Different letters indicate significant differences between different tissues (*P* ≤ .05, one-way ANOVA). (B) GUS staining of tomato fruit. Mature green and red-ripened fruits of two independent *SlLYK4p::GUS* transgenic lines and the wild type (WT) were cut in half and then stained with GUS solution for 24 hours. Representative images are shown. Scale bars: 0.5 cm. (C) Chitin induces *SlLYK4* expression in fruit. Mature green fruits were cut in half, soaked in H_2_O or chitin for 30 minutes, and then stained with GUS solution for 8 hours. Scale bars: 0.5 cm. (D, E) Disease assay of *B. cinerea* in fruit. Two holes were made with sterile needles in each fruit, and 2.5 μl of spore suspension (2 × 10^5^ spores/ml) was placed in the holes. Disease symptoms were observed 3 dpi. Representative images are shown in (D) and disease severity in (E). Severity of disease was graded from mild mycelia in the injection hole (first grade) to mycelia covering the fruit surface (third grade). Scale bars: 1 cm.

Stable transgenic *slcerk1* mutants were also generated using CRISPR/Cas9 technology. gRNA was designed at the 5′ end of *SlCERK1* and 10 nt from the start codon (Supplementary Data Fig. S6A). A 6-bp deletion and 1-bp insertion were found in *slcerk1-2* and *slcerk1-3* mutants, respectively (Supplementary Data Fig. S6A). Transcript levels of *SlCERK1* in both *slcerk1* mutant lines were significantly lower than those in the wild type (Supplementary Data Fig. S6B). Although the 1-bp insertion in *slcerk1-3* resulted in a premature stop codon at 87–89 nt*,* the transcript levels of *SlCERK1* were slightly higher in *slcerk1-3* than in *slcerk1-2*, probably because of the low efficiency of nonsense-mediated mRNA decay for premature stop codons within 200 nt of the start codon [48]. In addition, another start codon downstream at 169–171 nt might initiate a truncated version of SlCERK in *slcerk1-3.* Upon chitin treatment, ROS production and MAPK phosphorylation in *slcerk1-2* and *slcerk1-3* were lower than those in the wild type, but *slcerk1-3* showed a stronger reduction than *slcerk1-2* (Supplementary Data Fig. S6C–E). Furthermore, compared with the wild type, *slcerk1* mutants were more susceptible to *B. cinerea* infection (Supplementary Data Fig. S6F–H). These results suggest that mutation of *SlLYK4* and *SlCERK1* impairs chitin-induced immune responses in leaves.

### SlLYK4 and SlCERK1 are chitin receptors in tomato

AtLYK5 kinase is inactive owing to the lack of the critical amino acid for ATP binding [32]. Therefore, we compared the intracellular kinase domain of SlCERK1 and SlLYK4 with AtLYK4, AtLYK5, and AtCERK1 (Supplementary Data Fig. S7). P-loop subdomain I, RD domain in subdomain VIa, and DFG domain in subdomain VII are generally regarded as essential for kinase activity [49]. Similar to AtLYK4 and AtLYK5, SlLYK4 lacks these critical amino acids, suggesting that SlLYK4 might be a kinase-inactive protein. To validate this hypothesis, the GST tag was fused to the cytosolic kinase domain of SlLYK4 (referred to as GST-SlLYK4^CD^), SlCERK1 (GST-SlCERK1^CD^), and the kinase-dead version of SlCERK1^CD^ (GST-SlCERK1^CD-K355E^). The purified proteins were used for the *in vitro* kinase assay. GST-SlCERK1^CD^ proteins showed strong autophosphorylation, whereas GST-SlCERK1^CD-K355E^ and GST-SlLYK4^CD^ had no kinase activity (Fig. 3A). In addition, GST-SlCERK1^CD^ can transphosphorylate GST-SlLYK4^CD^ (Fig. 3A). These results suggest that SlLYK4 is a kinase-inactive protein.

To detect the binding affinity of SlLYK4 and SlCERK1 to chitin, we fused them with a C-terminal GFP tag and transiently expressed them in *N. benthamiana*. As a transient expression of *SlCERK1* in *N. benthamiana* caused cell death in leaves, it was hard to extract proteins [46]. Therefore, a kinase-dead version of SlCERK1^K355E^ was used in this assay. Chitin-binding proteins were pulled down with chitin-magnetic beads. We found that SlLYK4 proteins were pulled down with chitin-magnetic beads while SlCERK1^K355E^ and SlLYK7 were not (Fig. 3B). These results suggest that SlLYK4 has a higher binding affinity to chitin compared with SlCERK1^K355E^. In *Arabidopsis*, chitin induces the association of AtLYK5 and AtCERK1 [32]. To investigate whether this mechanism is the same in tomatoes, we fused SlLYK4 and SlCERK1^K355E^ with HA and Myc tag, respectively, and transiently expressed them in *N. benthamiana*. Upon chitin treatment, SlLYK4-HA was co-immunoprecipitated with SlCERK1^K355E^-Myc (Fig. 3C), suggesting that chitin induces the heterodimerization of SlLYK4 and SlCERK1. Chitin-induced SlLYK4-SlCERK1 association was verified in *Arabidopsis* protoplasts via polyethylene glycol (PEG)-mediated transient expression (Supplementary Data Fig. S8A). SlLYK4 interacted with SlCERK1 at the plasma membrane, as demonstrated by the BiFC assay when SlLYK1^K355E^-YFPc and SlLYK4-YFPn were co-expressed in *Arabidopsis* protoplasts (Supplementary Data Fig. S8B). Collectively, these results suggest a conserved model of chitin recognition, in which SlLYK4 binds chitin, and then forms a heterodimer with SlCERK1 to transduce the signal into the cytosol.

### *SlLYK4* is highly expressed in fruit

We next examined whether *SlLYK4* and *SlCERK1* are expressed in tomato fruit. Therefore, we harvested various tomato organs containing roots, stems, leaves, flowers, and fruit at the mature green and red ripened stages from wild-type plants. We found that transcript levels of *SlLYK4* and *SlCERK1* were higher in fruits than in other organs; especially *SlLYK4* was highly expressed in mature green fruits (Fig. 4A and Supplementary Data Fig. S9). These results suggest that *SlLYK4* might be regulated at the transcript level in fruit; thus, we focus on SlLYK4 hereafter.

To confirm the spatial expression patterns of *SlLYK4*, we generated transgenic plants expressing GUS under the control of 1.5 kb *SlLYK4* promoters (*SlLYK4p::GUS* L6 and L12, respectively). GUS activity was measured in the leaves, stems, roots, flowers, and fruits 24 hours after GUS staining. Strong GUS activity, as indicated by the blue color, was observed in the mature green and red ripened fruits (Fig. 4B), whereas only weak GUS activity was detected in leaves and flowers (Supplementary Data Fig. S10A). Almost no GUS activity was observed in cotyledons, stems, and roots (Supplementary Data Fig. S10B). To reduce the background of GUS staining in fruits, we also stained the fruits with GUS solution for 8 h. We found that the blue color was still observed in fruit (Supplementary Data Fig. S10C), and chitin treatment induced GUS activity (Fig. 4C). These results suggest that *SlLYK4* might play a role in fruit resistance to the fungal pathogen by recognizing chitin.

### SlLYK4 contributes to fungal resistance in tomato fruit

To investigate whether *SlLYK4* is important for fruit resistance to the fungal pathogen, we inoculated red ripe-stage fruits of wild-type and *sllyk4* mutants with *B. cinerea*. Sterile needles were used to make a small hole in the fruit epidermis, and then 2.5 μl of spore suspension solution (2 × 10^5^ spores/ml) of *B. cinerea* was pipetted into each hole. The disease incidence was scored 3 dpi on a scale from 1 to 3 according to the following criteria: (1) the inoculated wounds showed slight discoloration but had no fungal mycelia; (2) the inoculated wounds were slightly rotten and contained some mycelia; (3) the inoculated wounds were covered with mycelia, and the fruit displayed extensive water soaking and necrosis. Compared with the wild type, the fruits of *sllyk4* mutants were more susceptible to *B. cinerea* (Fig. 4D and E). These results suggest that *SlLYK4* is important in fruit resistance to *B. cinerea*.

Various postharvest physical and chemical treatments have been applied to reduce fungal infection and maintain fruit quality [50–52]. Therefore, we examined whether these treatments regulated the expression of *SlLYK4* in tomato fruit, such as low temperature, sodium chloride (NaCl), calcium chloride (CaCl_2_), and mannitol. Treatments with CaCl_2_ and low temperature significantly induced *SlLYK4* expression, but NaCl and mannitol did not (Supplementary Data Fig. S11). Overall, these results suggest that *SlLYK4* is highly expressed and regulated in tomato fruit, thus providing an idea to enhance chitin-mediated basal defense in tomato by overexpression of *SlLYK4* gene.

### *SlLYK4* overexpression renders plants more resistant to *B. cinerea*

**Figure 5:**
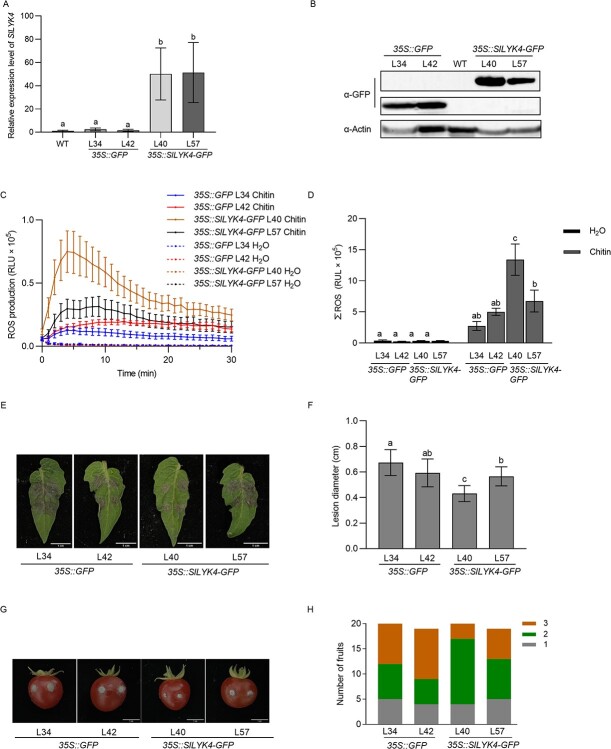
SlLYK4 overexpression renders plants more resistant to *B. cinerea.* (A) Relative expression levels of *SlLYK4* in ‘Micro-Tom’ (WT) and *35S::GFP* and *35S::SlLYK4-GFP* transgenic plants. RNA was extracted from 2-week-old leaves and roots, and gene expression was quantified using qRT–PCR. *SlEF1α* was used as an internal control. Data are presented as the mean ± standard deviation (*n* = 3). Different letters indicate significant differences between genotypes (*P* ≤ .05, one-way ANOVA). (B) Protein levels in transgenic plants were determined via immunoblotting using an anti-GFP antibody. Actin was the loading control. (C, D) Production of ROS after treatment with 10 μg/ml chitin. ROS were measured in *35S::SlLYK4-GFP* and *35S:GFP* transgenic plants. Total ROS are shown in (D). The line graphs are shown in (C). Data are expressed as mean ± standard error (*n* = 6). Different letters indicate significant differences between genotypes (*P* ≤ .05, one-way ANOVA). (E, F) Disease assay of *B. cinerea* in leaves. Leaves were spot-inoculated with *B. cinerea* spores (1 × 10^5^ spores/ml). Disease symptoms were observed 3 dpi. Representative images are shown in (E). Lesion diameter is shown in (F). Data are presented as mean ± standard deviation (*n* = 10). Different letters indicate significant differences between genotypes (*P* ≤ .05, one-way ANOVA). (G, H) Disease assay of *B. cinerea* in fruit. Fruits were hole-inoculated with *B. cinerea* spores (2 × 10^5^ spores/ml). Disease symptoms were observed at 3 dpi. Representative images are shown in (G) and disease severity is shown in (H). The severity of the disease was graded from mild mycelia in the injection hole (first grade) to mycelia covering the fruit surface (third grade). Scale bars: 1 cm.

To generate *SlLYK4* overexpression plants, the coding sequence of *SlLYK4* was fused to *GFP* and driven by the constitutive CaMV 35S promoter, referred to as *35S::SlLYK4-GFP*, whereas a *35S::GFP* construct was used as negative control. Two independent lines for each construct were chosen for further analysis. Although the transcript levels of *SLYK4* showed no significant differences between the two *35S::SlLYK4-GFP* transgenic lines (Fig. 5A), the protein levels of SlLYK4-GFP in *35S::SlLYK4-GFP* L40 were higher than in L57 plants (Fig. 5B). Consistent with the protein levels, chitin-induced ROS production in L40 plants was significantly higher than in L57, as well as higher than in the control *35S::GFP* plants (Fig. 5C and D). SlLYK4-GFP proteins were located at the cell peripheral region (Supplementary Data Fig. S12). When plant leaves were inoculated with *B. cinerea*, *35S::SlLYK4-GFP* L40 leaves barely developed disease symptoms. In contrast, the control *35S::GFP* leaves were almost covered by *B. cinerea* mycelia and typical chlorotic necrosis (Fig. 5E and F). Similarly, the fruit of *35S::SlLYK4-GFP* L40 showed enhanced resistance to *B. cinerea* infection (Fig. 5G and H). In addition, *35S::SlLYK4-GFP* L40 leaves also exhibited enhanced resistance to *S. sclerotiorum* infection (Supplementary Data Fig. S13). Our results suggest that *SlLYK4* overexpression increases fungal resistance in leaves and fruit.

### Mutation and overexpression of *SlLYK4* do not affect fruit development

**Figure 6:**
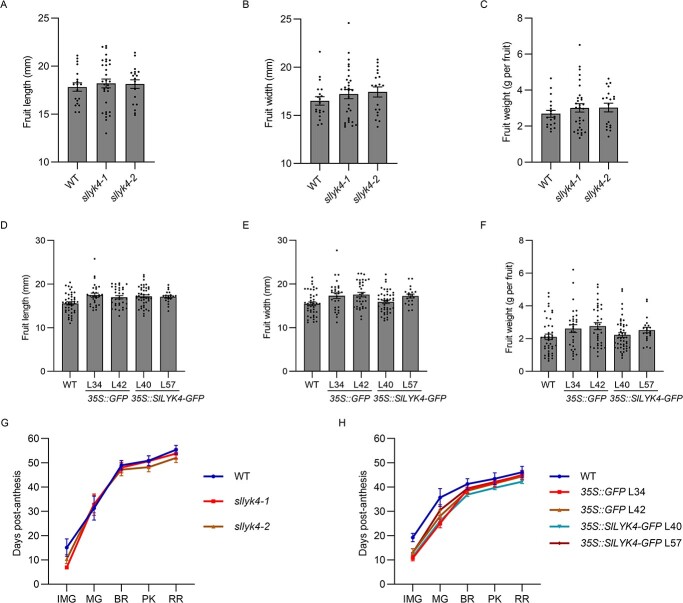
Mutation and overexpression of *SlLYK4* do not affect fruit development. (A–C) Fruit length (A), width (B), and weight (C) of the wild type (WT) and different *sllyk4* mutant lines. Values are mean ± standard error of 10 independent plants grown under the same conditions, and 18–22 red-ripened fruits were examined. (D–F) Fruit length (D), width (E), and weight (F) of WT, *35S::GFP*, and *35S::SlLYK4* overexpressing lines grown under the same conditions. Values are mean ± standard error of 10 independent plants grown under the same conditions, and 18–22 red-ripened fruits were examined. (G, H) Number of days from flowering to each stage of fruit development in WT and different *sllyk4* mutant lines (G) and *35S::GFP* and *35S::SlLYK4-GFP* (H) overexpressing lines. All plants were grown under the same conditions. Each error bar represents the mean ± standard error (*n* = 14).

Overexpression of PRRs often induces constitutive immunity, leading to a growth penalty. Therefore, we measured the fruit traits in the wild type, *sllyk4* mutants, *35S::SlLYK4-GFP*, and *35S::GFP* plants planted under the same conditions. Fruit size (length and width) of all genotypes showed no significant differences (Fig. 6A–F). The weight of individual fruit in *sllyk4* mutants and *35S::SlLYK4-GFP* plants was the same as in their controls. The fruit development time from flowering to fruiting was similar in all genotypes (Fig. 6G and H). We also compared the cuticle thickness of red-ripened fruit and did not find significant differences between genotypes (Supplementary Data Fig. S14). Together, these results suggest that *SlLYK4* overexpression increases fungal resistance without impairing fruit development.

## Discussion

Chitin, a typical fungal cell wall PAMP, elicits plant defenses against fungal diseases [10–12]. In this study, we found that the chitin receptor, *SlLYK4*, was highly expressed in tomato fruit, and *SlLYK4* overexpression enhanced fruit resistance to *B. cinerea*. Importantly, *SlLYK4* overexpression did not impair fruit development. This study provides a rationale for breeding disease-resistant tomatoes by enhancing the chitin-induced immune responses.

Chitin-induced immunity can potentially improve crops with broad-spectrum and durable disease resistance, as chitin is a major element of fungal cell walls. Mutations of chitin receptors in many species have reduced resistance to different fungal infections. For example, *Atcerk1* and *Atlyk4*/*5* are more susceptible to *B. cinerea*, *Alternaria brassicicola*, *Fusarium oxysporum* f. sp. *cubense*, *F. oxysporum* f. sp. *vasinfectum*, *Verticillium dahlia*, and *Blumeria graminis* f. sp. *hordei* [34, 53–56]. In this study, *sllyk4* and *slcerk1* impaired chitin-induced immunity and increased susceptibility to *B. cinerea*. In contrast, *SlLYK4* overexpression yielded increased resistance to *B. cinerea* and *S. sclerotiorum*. Regarding universal chitin perception, it is reasonable to hypothesize that tomato plants with *SlLYK4* overexpression might be resistant to various fungal diseases and provide broad-spectrum and durable disease resistance.

Constitutive expression of PRRs enhances plant resistance but often causes growth trade-offs [57, 58]. However, *SlLYK4* overexpression enhanced tomato resistance to fungal disease but did not impair fruit development. One possible explanation is that SlLYK4 is not activated to induce downstream responses without association with SlCERK1 in normal growth conditions even when overexpressed, whereas after pathogen infection *SlLYK4* overexpression might considerably increase the association of SlLYK4 and SlCERK1, possibly leading to the activation of more SlCERK1. Therefore, *SlLYK4* overexpression does not cause autoimmunity under normal growth conditions but increases resistance after infection with the fungal pathogen. These findings provide a possible mechanism that improves inducible defense responses by overexpression of the receptor-like proteins or inactive receptor-like kinases, which will only mediate increased immune responses upon pathogen infection.

In the case of *Arabidopsis*, chitin is recognized by AtLYK4/LYK5 and then induces the association with AtCERK1, leading to AtCERK1 activation to transduce the signal from the membrane to the cytosol [32]. In this study, in tomato, SlLYK4 binds to chitin with higher affinity than SlCERK1, and chitin stimulates SlLYK4 association with SlCERK1, further activating the cytoplasmic signaling events. Our studies revealed a conserved chitin recognition model between *Arabidopsis* and tomato. Among SlLYK4, SlLYK6, and SlLYK7, only SlLYK4 silencing showed reduced chitin-induced ROS production; however, we cannot rule out the possibility that SlLYK6 and SlLYK7 play a minor role in chitin recognition because *sllyk4* mutants did not completely abolish chitin responses. In addition, we found that SlCERK1 can phosphorylate SlLYK4, similar to the *Arabidopsis* mechanism, but different from the observation in cotton that GhCERK1 cannot phosphorylate GhLYK5 *in vitro* [34]. In addition, SlLYK4 is highly expressed in tomato fruit, but AlLYK5 and GhLYK5 did not show this pattern (http://bar.utoronto.ca/eplant/ and http://cotton.zju.edu.cn/). Overall, the transcriptional regulation of *SlLYK4* in fruit is worth further study.

Postharvest diseases in fruit cause considerable economic losses [59]. Therefore, efforts have been made to develop non-fungicidal methods to control postharvest fruit decay, including low temperature and treatment with safe chemicals [60]. In this study, low temperature highly induced SlLYK4 expression in fruit, suggesting that low temperature not only delays pathogen growth but also enhances fruit immunity. In addition, CaCl_2_ treatment, in combination with many antagonists, improves efficiency in controlling post-harvest diseases [61]. In this study, *SlLYK4* expression was slightly upregulated after CaCl_2_ treatment, and this partially explains why treatment with CaCl_2_ improves the efficiency of antagonists. As *SlLYK4* expression in fruit is regulated by many factors, it is worth screening other factors, e.g. light, which might induce *SlLYK4* expression to enhance disease resistance.

Overall, we found a conserved chitin recognition model between *Arabidopsis* and tomato. Unlike *Arabidopsis*, tomato chitin receptor *SlLYK4* is highly expressed in fruit, and *SlLYK4* overexpression renders tomato fruit more resistant to gray mold disease. Our study provides a potential strategy to enhance tomato fruit resistance via modifying *SlLYK4* expression.

## Materials and methods

### Plant materials and growth conditions

CRISPR-Cas9 mutants and transgenic overexpression lines were generated in *S. lycopersicum* cv. ‘Micro-Tom’, which is a model cultivar for genetic transformation. For virus-induced gene silencing (VIGS) experiments, *S. lycopersicum* cv. ‘Zheza 809’ was used due to its high silencing efficiency. Seeds were sterilized with 75% ethanol for 30 seconds, washed with sterilized water, then with 10% NaClO for 10 minutes, and washed with sterilized water. Seeds were grown on ½-strength Murashige and Skoog agar plates under a 16-hour photoperiod, 75% humidity, and a temperature of 25°C for 10 days, then transferred to soil containing peat-based compost and grown in a greenhouse with the same photoperiod and temperature.

### Plasmid construction and generation of transgenic plants

The primers used for gene cloning are listed in Supplementary Data Table S1. Full-length coding sequences or fragments were amplified from cDNA. The amplified sequences were cloned into the pDONR/Zeo plasmid by BP cloning (Invitrogen, Waltham, MA, USA). After verification by sequencing, the resultant plasmids were cloned into the destination vectors (Supplementary Data Table S2) by LR cloning (Invitrogen). Tomato transgenic plants were generated as previously described [6].

### Phylogenetic analysis and protein domain prediction

The full-length amino acid sequences were used to construct an unrooted neighbor-joining phylogenetic tree using MEGA 6.0 software. The bootstrap test was replicated 1000 times. The amino acid sequences of SlLYK and AtLYK proteins were acquired from the Sol genomics network (https://solgenomics.net/) and TAIR databases (https://www.arabidopsis.org), respectively. The accession numbers of genes are listed in Supplementary Data Table S3. The domains of SlCERK1 and SlLYK4 were predicted using InterPro (https://www.ebi.ac.uk/interpro/).

### Virus-induced gene silencing assay

VIGS was performed as described by Wang *et al.* [6]. Fragments of *SlLYK4*, *SlLYK6*, and *SlLYK7* were inserted into the tobacco rattle virus 2 (TRV2) vector. pTRV2-*GUS* (*β-GLUCURONIDASE*) served as a negative control. pTRV2-*PDS* (*PHYTOENE DESATURASE*) was used to monitor the progress of gene silencing.

### RNA isolation and quantitative real-time PCR

Total RNA was extracted using an Easy Plant RNA Extraction Kit (Easy-Do Biotech, Hangzhou, China). First-strand complementary DNA was synthesized from 1 μg of RNA using HiScript II reverse transcriptase (Vazyme Biotech, Nanjing, China). SYBR Green Master Mix was used in the qRT–PCR reactions (Vazyme Biotech). The relative levels of gene expression were calculated using the 2^-∆∆Ct^ method. *SlELONGATION FACTOR1α* (*SlEF1α*, Solyc06g005060) and *SlACTIN7* (Solyc03g078400) served as internal controls. All primers used for the qRT–PCR are listed in Supplementary Data Table S1.

### Chemiluminescence assay for reactive oxygen species detection

A chemiluminescence assay was used to detect ROS production. Leaf disks (diameter, 0.5 cm) were incubated in water overnight. After adding 1.25 μM L-012 chemiluminescent probe (Wako Chemicals USA, Richmond, VA, USA), 20 μg/mL horseradish peroxidase, and 50 μg/mL chitin mixture (Merck, Darmstadt, Germany), chemiluminescent signals were immediately recorded using a Photek camera (HRPCS5; Photek, East Sussex, UK).

### Transient expression in *Nicotiana benthamiana*

Plasmids were electroporated into the *Agrobacterium tumefaciens* strain GV3101, and then the resulting bacteria were infiltrated into *N. benthamiana* leaves according to a previously described method [6]. Proteins were extracted 48 hours after infiltration.

### Protein extraction and immunoblot assay

Total proteins were extracted using extraction buffer containing 50 mM Tris–HCl (pH 7.5), 150 mM NaCl, 2% Triton X-100, and 1× Protease Inhibitor Cocktail. To detect MAPK phosphorylation, 1 nM calyculin A and 25 mM NaF were added to the extraction buffer. The green fluorescent protein (GFP) was detected using an anti-GFP antibody (Miltenyi Biotec, Bergisch Gladbach, Germany), and phosphorylation of MAPK was detected using an anti-p44/42 MAPK antibody (Cell Signaling Technology, Danvers, MA, USA). Anti-actin antibody (ABclonal Biotechnology, Wuhan, China) served as a loading control [62].

### *In vitro* kinase assay

*In vitro* kinase assays were performed as described previously [63]. The cytosolic domains of SlCERK1 and SlLYK4 were fused with a glutathione-*S*-transferase (GST) tag at their N-termini and expressed in *Escherichia coli*. Kinase assays were performed with purified proteins, and protein phosphorylation was detected with an immunoblot analysis using an anti-pThr/Ser antibody (Cell Signaling Technology).

### *Arabidopsis* protoplast transformation

*Arabidopsis* protoplast transformation was performed as previously described by Yoo *et al.* [64]. For bimolecular fluorescence complementation (BiFC) assay, 200 μl protoplasts of ~2 × 10^5^ cells were transfected with 20 μg plasmids. After incubation in a growth chamber at 23°C overnight for ~14 hours, the transfected protoplasts were used to monitor the fluorescence signal. For the co-immunoprecipitation (Co-IP) assay, 1 ml protoplasts of ~10^6^ cells were transfected with 100 μg plasmids, and after incubation were treated with 100 μg/ml chitin for 15 minutes.

### Co-immunoprecipitation

Total proteins were extracted from protoplasts and *N. benthamiana* leaves, and immunoprecipated with anti-hemagglutinin (HA) Magnetic Beads (MedChemExpress, NJ, USA). The co-immunoprecipated proteins were detected with an immunoblot assay using anti-Myc and anti-HA antibodies (Miltenyi Biotec).

### Chitin-binding assay

Total proteins were extracted from *N. benthamiana* leaves. Chitin-binding proteins were pulled down using chitin magic beads according to the manufacturer’s protocol (New England Biolabs, Ipswich, MA, USA) and then detected with an immunoblot assay using an anti-GFP antibody (Miltenyi Biotec).

### β-Glucuronidase histochemical staining

Tissues were stained with GUS solution [0.15 M NaH_2_PO_4_, pH 7, 2 mM K_4_Fe(CN)_6_, 2 mM K_3_Fe(CN)_6_, 0.05% Triton X-100, and 0.5 mM X-gluc]. After incubation at 37°C for 8 or 24 hours, tissues were cleared in 100% ethanol.

### Cuticle staining

Cuticle staining was performed as previously described [65]. Briefly, 8-μm sections of pericarp from red ripened fruits were stained with 0.06% Sudan IV for 10 minutes. Images were taken under a light microscope (Eclipse Ni-U; Nikon, Tokyo, Japan). Cuticle thickness was measured using ImageJ.

### Detection of green fluorescent protein

GFP from *35S::GFP* and *35S::SlLYK4-GFP* transgenic plants was observed under a confocal microscope (LSM 800; Zeiss, Oberkochen, Germany) using a 488-nm filter.

### Fungal inoculation

For *B. cinerea* inoculation, spores were collected using a suspension solution (4% maltose and 1% peptone) and then passed through two cheesecloth layers. Spore density was adjusted to 1 × 10^5^ spores/ml for leaf inoculation and 2 × 10^5^ spores/ml for fruit inoculation. For leaf inoculation, 2.5 μl of spore solution was dropped on the detached, fully expanded leaves. For fruit inoculation, a 1-cm deep hole was made in the fruit with a sterile syringe, and 2.5 μl of spore solution was added into the hole. Inoculated leaves and fruit were covered with a transparent plastic film and kept at 23°C in a growth chamber. The diameters of each lesion were recorded 3 days post-inoculation (dpi). The *B. cinerea* biomass was determined by DNA-based qPCR using primers for *Bc3* [66].

Inoculation of *Sclerotinia sclerotiorum* UF1 was performed as previously described [67, 68]. Fresh sclerotia were grown on potato dextrose agar (PDA) medium at 23°C. When the mycelium was about to contact the edge of the Petri dish, 5-mm diameter fungal plugs containing the young mycelia were punched and placed on 6-week-old tomato leaves. Inoculated leaves were covered with a transparent plastic film and kept at 23°C in a growth chamber. The area of each disease lesion was measured at 36 hours post-inoculation. For disease resistance evaluation, at least six plants for each genotype were examined.

## Acknowledgements

We thank Xiao-xiao Feng from the agricultural experiment station of Zhejiang University for her assistance with plant growth and Wen Wang from the Institute of Biotechnology of Zhejiang University for her help with the phylogenetic analysis. This work was supported by the Key Research and Development Program of Zhejiang Province (2021C02009, 2021C02064-7, and 2022C02016), the National Natural Science Foundation of China (31770263 and 31970279), and the National Key Research and Development Program of China (2018YFD1000800).

## Author contributions

Y.A. conducted most of the experiments and analyzed the data. Q.L., C.L., and R.W. helped with the tomato transformation. X.S. helped with the VIGS experiment. S.C. and X.C. helped with *Sclerotinia* inoculation. Y.A., X.Q., and Y.L. prepared the manuscript. All authors have read and approved the final manuscript.

## Data availability

The data and materials used to support the findings of this study are available from the corresponding author upon request.

## Conflict of interest

The authors declare that they have no conflict of interest.

## Supplementary data

Supplementary data is available at *Horticulture Research* online.

## Supplementary Material

Web_Material_uhad082
