# Supplementary material for: Tomato LysM receptor kinase 4 mediates chitin-elicited fungal resistance in both leaves and fruit
Source: Hortic Res. 2023 Apr 25;10(6):uhad082. doi: 10.1093/hr/uhad082 (PMC10266952; doi:10.1093/hr/uhad082)
Supplement: Web_Material_uhad082 [file web_material_uhad082.pdf]

**Supporting Information**

Article title: **Tomato LysM receptor kinase 4 mediates chitin-elicited fungal resistance in both leaves and fruit**

Authors: Yingfei Ai[1], Qinghong Li[1], Chenying Li[1], Ran Wang[1], Xun Sun[1], Songyu Chen[1], Xin-Zhong Cai[2], Xingjiang Qi[3#], and Yan Liang[1#]

The following Supporting Information is available for this article:

**Fig. S1 Phylogenetic tree of LYK family in *Arabidopsis thaliana* and *Solanum lycopersicum*.**

**Fig. S2 Chitin induces *SlLYK4* expression.**

**Fig. S3 The transcript levels of *SlWRKY33* and *SlWRKY53* are lower in *sllyk4* mutants than in the wild type after chitin treatment.**

**Fig. S4 Biomass of *Botrytis cinerea* in *sllyk4* leaves is higher than in the wild type.**

**Fig. S5 *Sllyk4* mutants are susceptible to *Sclerotinia sclerotiorum*.**

**Fig. S6 *Slcerk1* mutants impair chitin-induced immune responses in tomato leaves.**

**Fig. S7 Sequence alignment of the kinase domain.**

**Fig. S8 SlLYK4 associates with SlCERK1.**

**Fig. S9 *SlCERK1* expression pattern.**

**Fig. S10 Spatiotemporal expression pattern of *SlLYK4*.**

**Fig. S11 *SlLYK4* expression in fruit is induced by low temperature and $CaCl_2$ treatment.**

**Fig. S12 SlLYK4 localizes to the cell peripheral region.**

**Fig. S13 *SlLYK4* overexpression enhances plant resistance to *Sclerotinia sclerotiorum*.**

**Fig. S14 *SlLYK4* overexpression does not affect fruit cuticle thickness.**

**Table S1** Primers used in this study

**Table S2** Vectors used in this study

**Table S3** Accession number of genes mentioned in this study

**Fig. S1 Phylogenetic tree of LYK family in *Arabidopsis thaliana* and *Solanum lycopersicum*.** An unrooted phylogenetic tree was constructed using the Maximum Likelihood method with the MEGA 6.0 program. The full-length amino acid sequences of all lysin motif receptor-like kinases (LYKs) from *A. thaliana* (At) and *S. lycopersicum* (Sl) were used for the phylogenetic tree. Robustness of the topology was assessed by 1000 bootstrap replications.

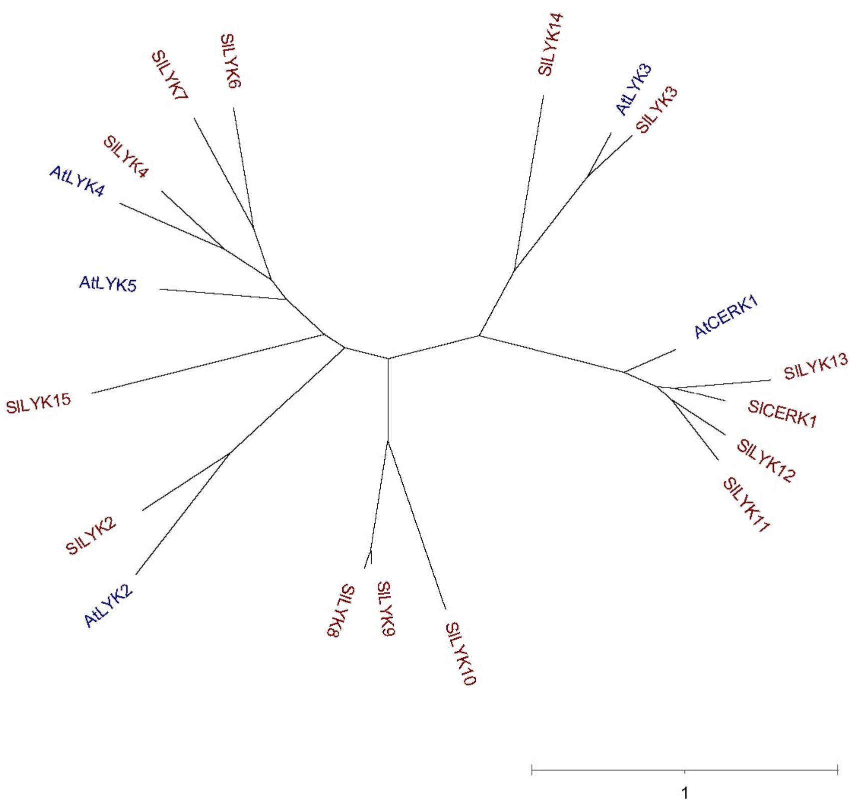

**Fig. S2 Chitin induces *SlLYK4* expression.** RNA was extracted from 2-week-old tomato roots (A) and leaves (B) 30 min after treatments with $H_2O$ or chitin. The transcript levels of *SlLYK4*, *SlLYK6*, and *SlLYK7* were detected by qRT-PCR. Data are expressed as mean ± SD (n = 3). *SlEF1α* served as the internal control. Asterisks indicate significant differences from the $H_2O$ control (**$P \leq 0.01$, Student's *t*-test). Ns stands for no significant differences.

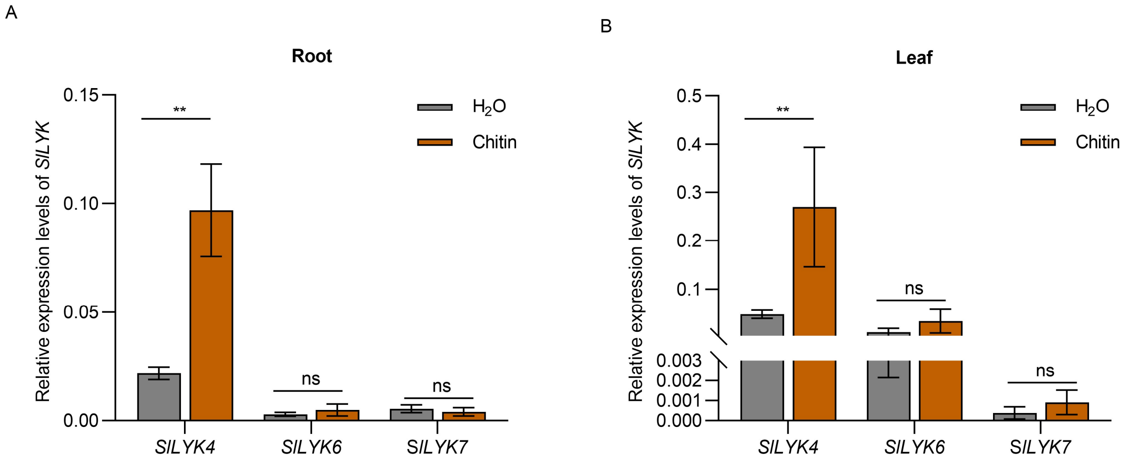

**Fig. S3 The transcript levels of *SlWRKY33* and *SlWRKY53* are lower in *sllyk4* mutants than in the wild type after chitin treatment.** RNA was extracted from 2-week-old tomato leaves of wild type (WT) and *sllyk4* mutants 30 min after treatments with $H_2O$ or chitin. The transcript levels of *SlWRKY33* (A) and *SlWRKY53* (B) were detected by qRT-PCR. *SlEF1α* was used as an internal control. Data are expressed as mean ± SD (n = 3). Different letters indicate significant differences between WT and *sllyk4* mutants ($P \leq 0.05$, one-way ANOVA).

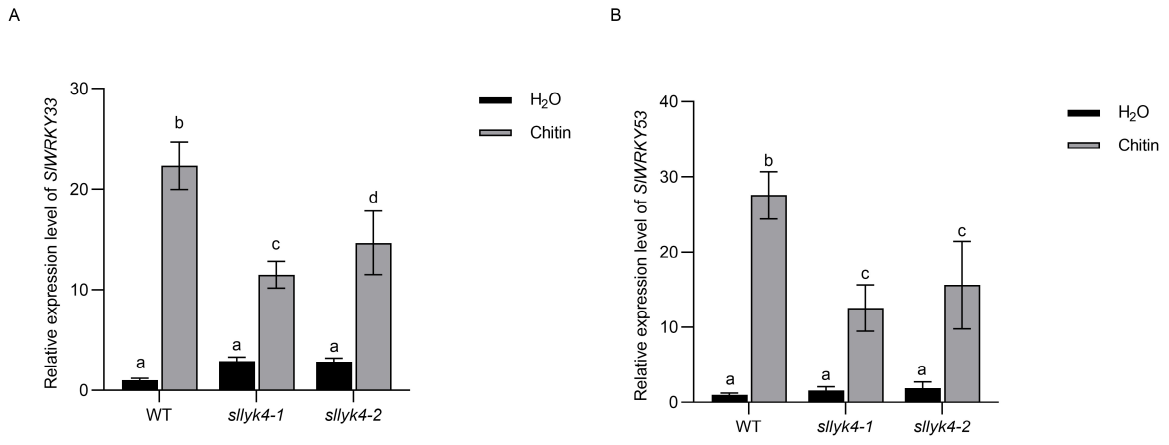

**Fig. S4 Biomass of *Botrytis cinerea* in *sllyk4* leaves is higher than in the wild type.** Six-week-old leaves were detached and drop-inoculated with 2.5 µL of *B. cinerea* spore suspension solution (1 × $10^5$ spores/mL). Biomass of *B. cinerea* was quantified by DNA-based qPCR. The plant biomass was normalized by *SlEF1α*. Data are expressed as mean ± SD (n = 4). Different letters indicate significant differences between wild-type (WT) and *sllyk4* mutants ($P \leq 0.05$, one-way ANOVA).

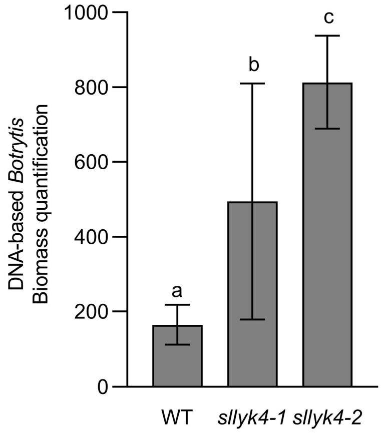

**Fig. S5 *Sllyk4* mutants are susceptible to *Sclerotinia sclerotiorum*.** Six-week-old detached leaves were inoculated with *S. sclerotiorum* **(A)**, and lesion areas ($cm^2$) were calculated 36 h post-inoculation **(B)**. Scale bars: 1 cm. Data are expressed as mean ± SE (n = 10). Different letters indicate significant differences between genotypes ($P \leq 0.05$, one-way ANOVA).

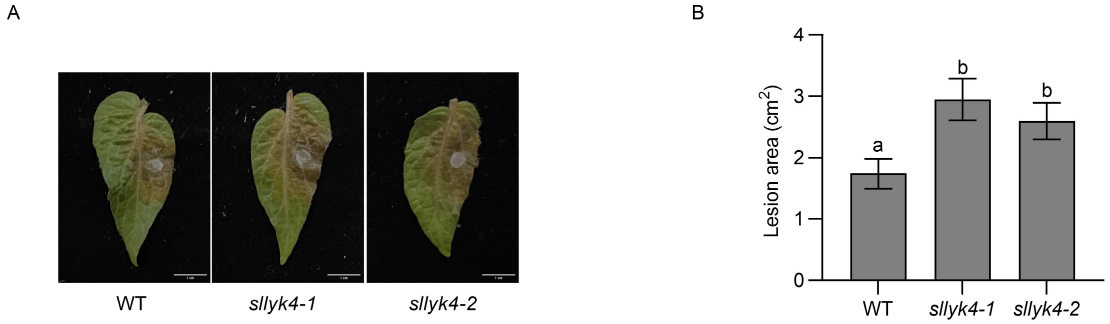

**Fig. S6 *Slcerk1* mutants impair chitin-induced immune responses in tomato leaves. (A)** Schematic representation of *SlCERK1*. Boxes and lines represent gene exons and introns; the gRNA sequences are shown above it, and the structural feature of the protein is shown below it. LysM, LysM motif; TM, transmembrane region (237–260 amino acids). The *slcerk1-2* line has a 6-bp deletion, and the *slcerk1-3* line has a 1-bp insertion. **(B)** The transcript levels of *SlCERK1* in *slcerk1-2* and *slcerk1-3* mutants were lower than those in the wild type (WT). RNA was extracted from 2-week-old WT and *slcerk1* mutant leaves and roots. The transcript levels of *SlCERK1* were detected by qRT-PCR. *SlEF1α* was used as an internal control. Data are presented as means ± SD from four biological replicates. Different letters indicate significant differences between WT and *slcerk1* mutants ($P \leq 0.05$, one-way ANOVA). **(C, D)** The *slcerk1* mutants showed reduced ROS production after chitin treatment (50 μg/mL). ROS levels were monitored using a chemiluminescence assay, and signals were recorded for 30 min. The line graphs were plotted with values recorded at 60-sec intervals (C), and the total ROS production is shown in (D). Data are presented as mean ± SE (n = 8). Different letters indicate significant differences between WT and *slcerk1* mutants ($P \leq 0.05$, one-way ANOVA). **(E)** The abundance of phosphorylated mitogen-activated protein kinase (MAPK). Proteins were extracted from 8-d-old tomato cotyledons at the indicated time points after treatment with 50 μg/mL chitin. MAPK phosphorylation was detected with an immunoblot analysis using an α-p42/44 MAPK antibody, and an α-Actin antibody served as a loading control. Band intensity was measured by Image J. Numbers on the blot indicate the relative levels of phosphorylated MAPK proteins in the mutants normalized to those in the WT. **(F-H)** The *slcerk1* mutants were susceptible to *B. cinerea*. Six-week-old tomato leaves were detached and spot-inoculated with 2.5 μL of *B. cinerea* spores ($1 \times 10^5$ spores/mL). Images were taken 3 d post inoculation (dpi), and representative images are shown in (F). Lesion diameter in leaves was measured (G). Data are presented as the means ± SD (n = 10). Biomass of *B. cinerea* was quantified by DNA-based qPCR (H). Data are presented as the means ± SD (n = 4). Different letters indicate significant differences between the WT and *slcerk1* mutants ($P \leq 0.05$, one-way ANOVA). Scale bars: 1 cm.

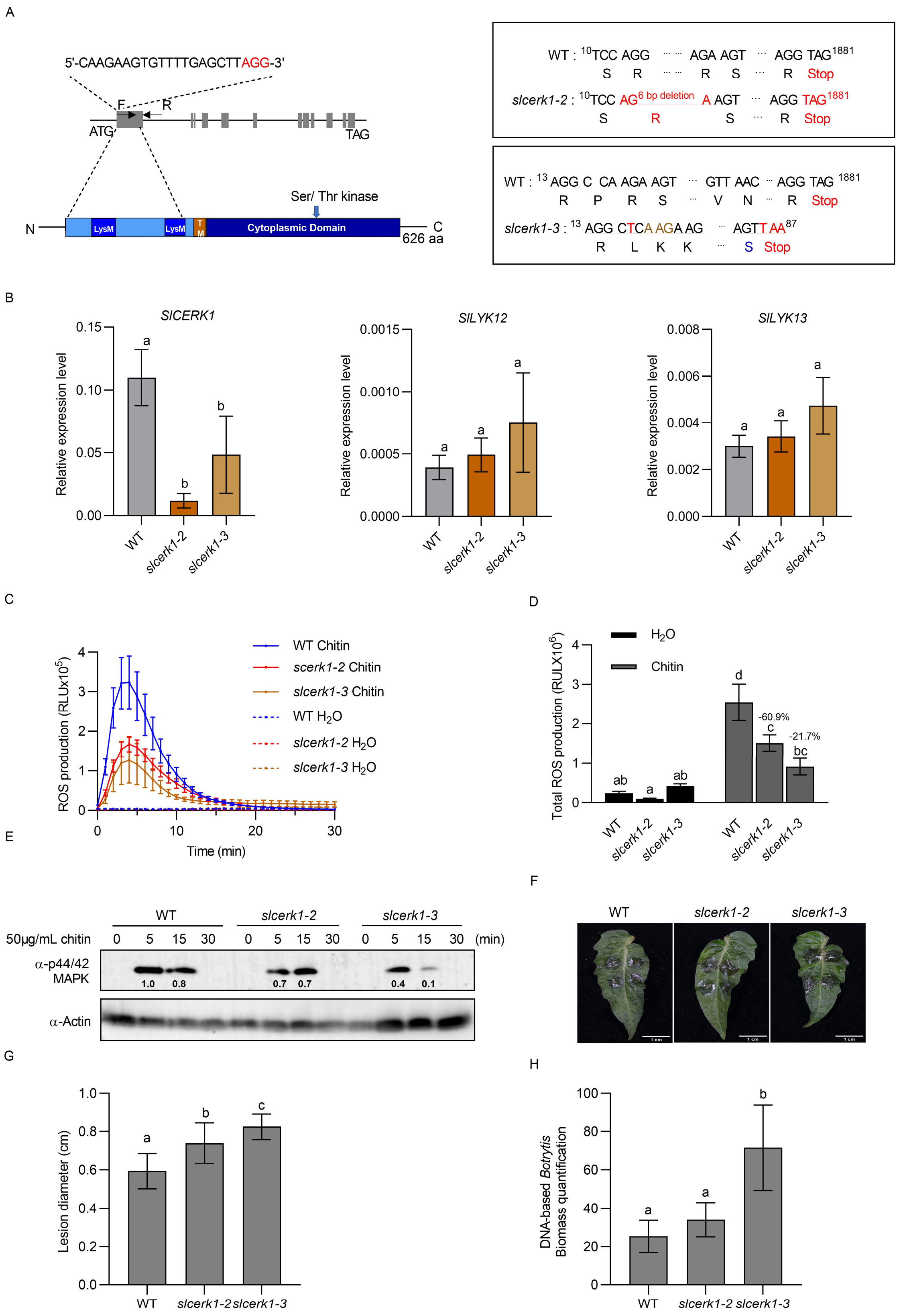
A
5'-CAAGAAGTGTTTTGAGCTTAGG-3'
F
R
ATG
TAG
N
LysM
LysM
TM
Cytoplasmic Domain
C
626 aa
Ser/ Thr kinase
WT : 10TCC AGG ... AGA AGT ... AGG TAG1881
S R R S ... R Stop
slcerk1-2 : 10TCC AG6 bp deletion A AGT ... AGG TAG1881
S R S ... R Stop
WT : 13 AGG C CA AGA AGT ... GTT AAC ... AGG TAG 1881
R P R S ... V N ... R Stop
slcerk1-3 : 13 AGG CTCA AGA AG ... AGTT AA87
R L K K ... S Stop
B
SlCERK1
SlLYK12
SlLYK13
Relative expression level
WT
slcerk1-2
slcerk1-3
C
ROS production (RLUx10^5)
Time (min)
WT Chitin
scerk1-2 Chitin
slcerk1-3 Chitin
WT H2O
slcerk1-2 H2O
slcerk1-3 H2O
D
Total ROS production (RULX10^6)
H2O
Chitin
-60.9%
-21.7%
E
WT
slcerk1-2
slcerk1-3
50µg/mL chitin
0 5 15 30 0 5 15 30 0 5 15 30 (min)
α-p44/42 MAPK
1.0 0.8 0.7 0.7 0.4 0.1
α-Actin
F
WT
slcerk1-2
slcerk1-3
G
Lesion diameter (cm)
H
DNA-based Botrytis Biomass quantification

**Fig. S7 Sequence alignment of the kinase domain.** Multiple Sequence alignment was carried out by ClustalW using the amino acid (aa) sequences from the intracellular domain of SlLYK4, SlCERK1, AtCERK1, AtLYK4, and AtLYK5. The kinase subdomains are labeled with roman numerals. Red boxes represent amino acids necessary for kinase activity. Fragments used for alignment are shown as follows: SlLYK4 (Solyc02g089900): 294–645 aa, SlCERK1 (Solyc07g049180): 256–626 aa, AtLYK5 (At2g33580): 301–664 aa, AtLYK4 (At2g23770): 295–612 aa, AtCERK1 (At3g21630): 255–671 aa.

A

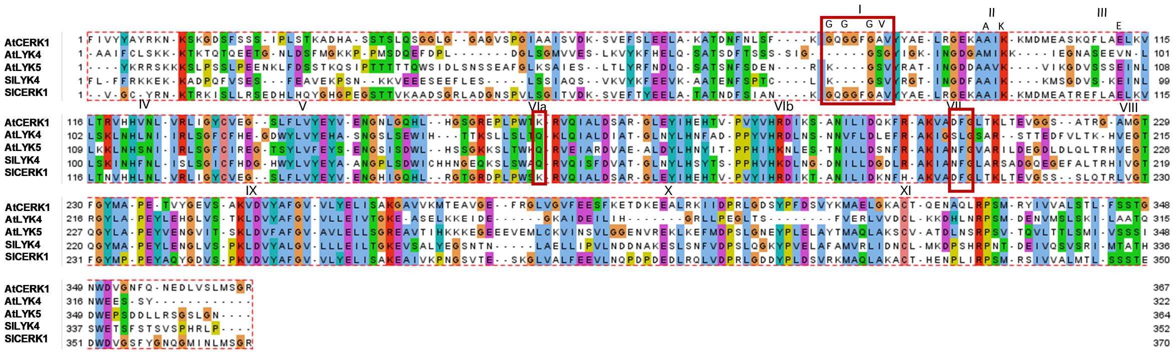

**Fig. S8 SlLYK4 associates with SlCERK1. (A)** Chitin-induced SlLYK4-SlCERK1 association. HA-tagged SlLYK4 and Myc-tagged SlCERK1 were co-expressed in *Arabidopsis* protoplasts. Proteins were extracted from protoplasts 15 min after treatment with or without chitin (100 μg/mL) and then immunoprecipitated with α-HA magnetic beads. Input and co-immunoprecipitated proteins were detected by immunoblot analysis with α-HA and α-Myc antibodies. **(B)** SlLYK4 interacted with SlCERK1 at the plasma membrane. SlLYK4-SlCERK1 association was detected by a bimolecular fluorescence complementation assay. SlLYK4 and SlCERK1 were fused to the N- or C-terminal portion of the yellow fluorescence protein (YFP). The fusion proteins were co-expressed in *Arabidopsis* protoplasts. Images were obtained using a confocal laser scanning microscope.

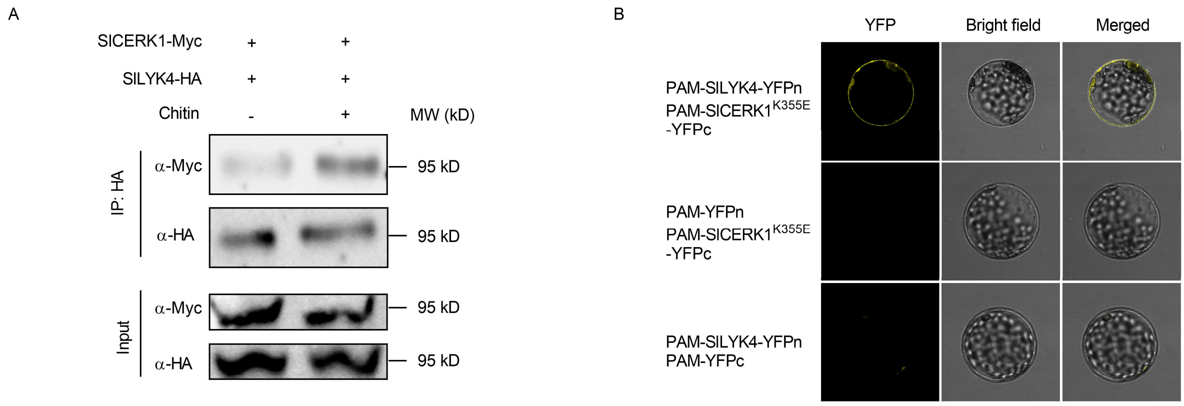

**Fig. S9 *SlCERK1* expression pattern.** RNA was extracted from the root, stem, leaf, flower, and fruit of tomato Micro-Tom. Transcript levels of *SlCERK1* were detected using qRT-PCR. *SlEF1α* was used as an internal control. Data are presented as the means ± SD (n = 3). Different letters indicate significant differences in tissues ($P \leq 0.05$, one-way ANOVA).

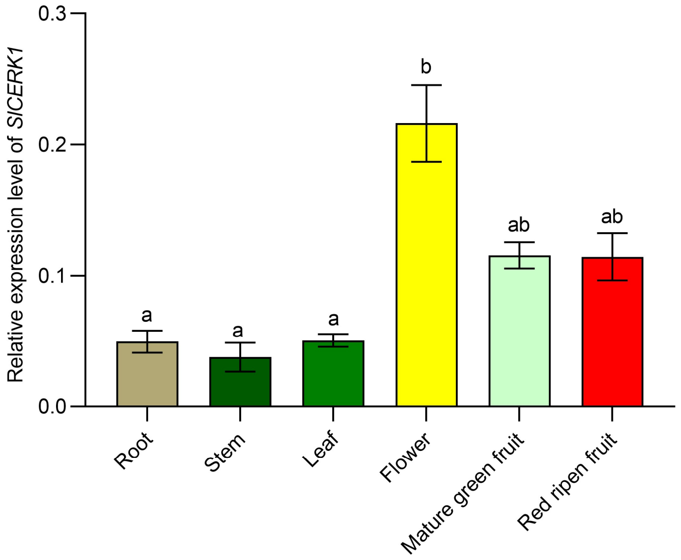

**Fig. S10 Spatiotemporal expression pattern of *SlLYK4*. (A)** Histochemical GUS staining of floral organs, root, stem, and leaf of *SlLYK4p::GUS* transgenic tomato plants for 24 h. Scale bars: 0.5 cm. **(B)** Histochemical GUS staining of 8-d-old transgenic tomato seedlings for 24 h. Scale bars: 200 μm. **(C)** Histochemical GUS staining of tomato immature green fruit, mature green fruit, and breaker stage fruit for 8 h. Scale bars: 0.5 cm.

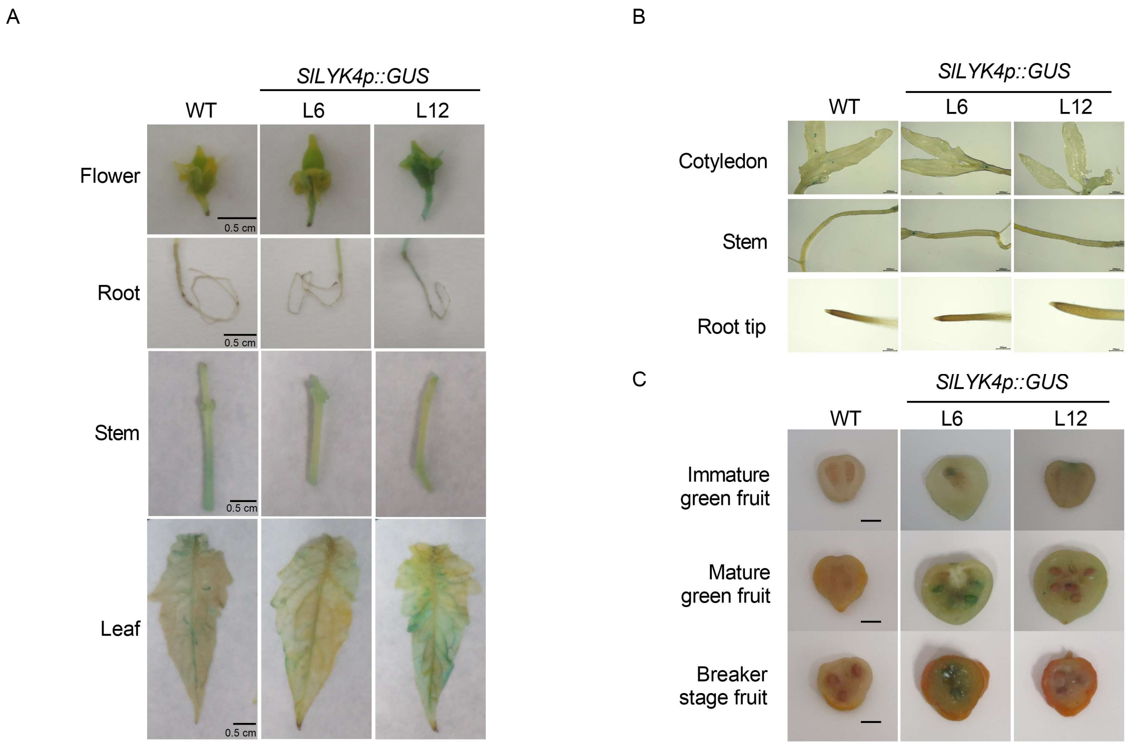

**Fig. S11 *SlLYK4* expression in fruit is induced by low temperature and $CaCl_2$ treatment.** RNA was extracted from fruit after treatments with low temperature (A), 1% $CaCl_2$, 2% NaCl, and 500 mM mannitol (B). The transcript level of *SlLYK4* was detected by qRT-PCR. *SlACTIN7* was used as an internal control. Data are presented as means ± SD (n = 3). Asterisks indicate significant differences from the control treatment (*$P \leq 0.05$, Student's *t*-test). Ns stands for no significant differences.

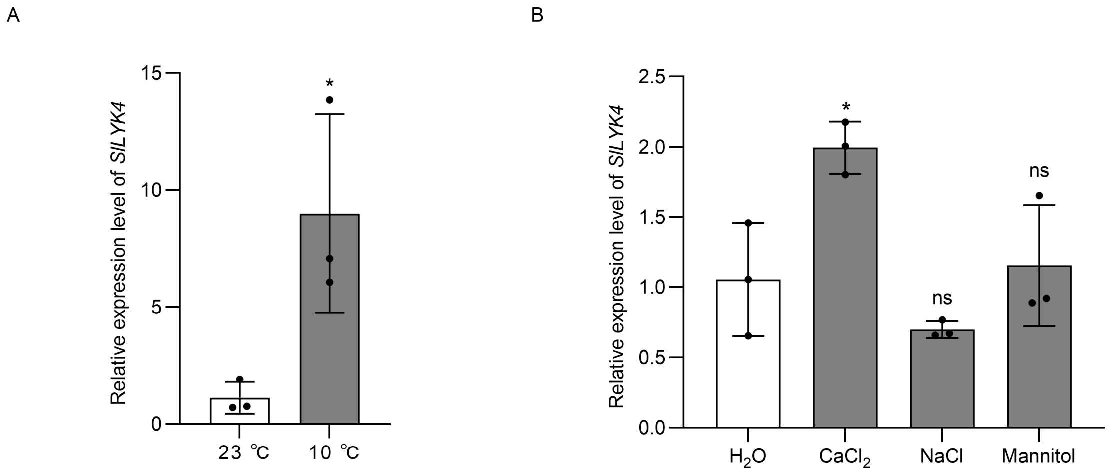

**Fig. S12 SlLYK4 localizes to the cell peripheral region.** Two-week-old leaves from *35S::GFP* (as control) and *35S::SlLYK4-GFP* transgenic lines were used to detect the subcellular localization of green fluorescent proteins. Images were obtained using a confocal laser scanning microscope. Scale bars: 10 µm.

A

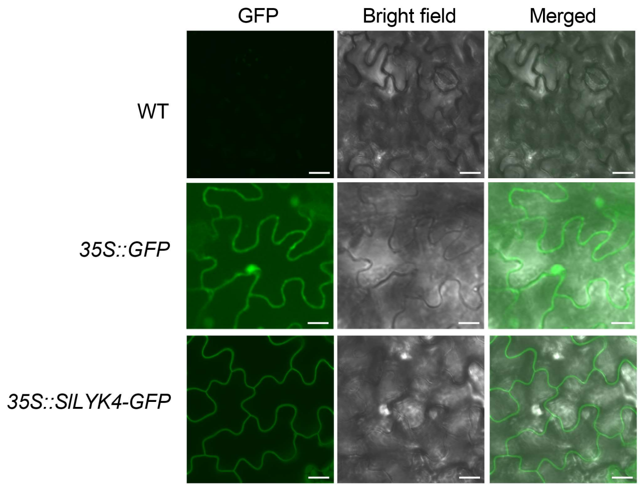

**Fig. S13 *SlLYK4* overexpression enhances plant resistance to *Sclerotinia sclerotiorum*.** Six-week-old detached leaves were inoculated with *S. sclerotiorum* **(A)**, and lesion areas (cm$^2$) were calculated 36 h post-inoculation **(B)**. Scale bars: 1 cm. Data are expressed as mean ± SE (n = 10). Different letters indicate significant differences between genotypes ($P \leq 0.05$, one-way ANOVA).

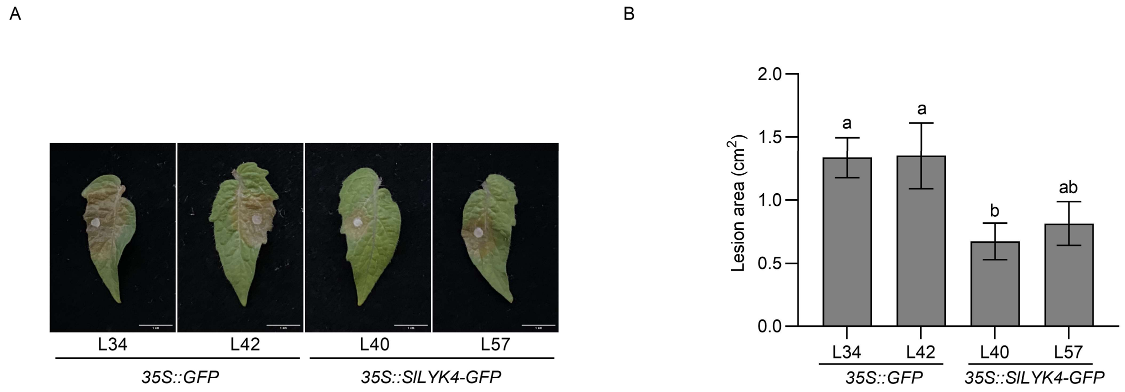

**Fig. S14 *SlLYK4* overexpression does not affect cuticle thickness. (A)** Cuticle sections stained with Sudan IV to visualize the cutinization of epidermal cell walls in Micro-Tom (WT), *35S::GFP*, and *35S::SlLYK4-GFP*. Scale bars: 25 µm. **(B)** Quantification of the cuticle thickness of epidermal cell walls. Different letters indicate significant differences between genotypes ($P \leq 0.05$, one-way ANOVA).

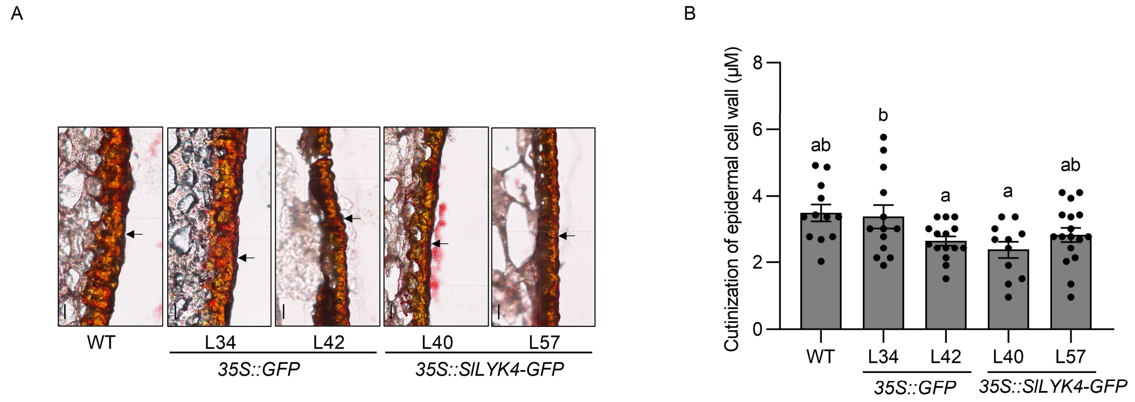

**Table S1** Primers used in this study

| Primers | Sequences (5'-3') |
|---|---|
| SlLYK4-GW-F | GGGGACAAGTTTGTACAAAAAAGCAGGCTACATGAATTATTCTCATCTCATC |
| SlLYK4-GW-R | GGGGACCACTTTGTACAAGAAAGCTGGGTCGGGCAATCTATGTGGTGACA |
| SlLYK6-GW-F | GGGGACAAGTTTGTACAAAAAAGCAGGCTACATGAATTGGCTTTTGAATATT |
| SlLYK6-GW-R | GGGGACCACTTTGTACAAGAAAGCTGGGTCGGTTGGTACTGCAGCAGAAA |
| SlLYK7-GW-F | GGGGACAAGTTTGTACAAAAAAGCAGGCTACATGGGTGATTTTCCACTTAT |
| SlLYK7-GW-R | GGGGACCACTTTGTACAAGAAAGCTGGGTCGTTTTGTACATCTGACTGGA |
| SlLYK4-VIGS-F | GGGGACAAGTTTGTACAAAAAAGCAGGCTACGATGATAATGCAAAGGAGAGTTTGAG |
| SlLYK4-VIGS-R | GGGGACCACTTTGTACAAGAAAGCTGGGTCTGGTGACACTGAAGTGCTAAAT |
| SlLYK6-VIGS-F | GGGGACAAGTTTGTACAAAAAAGCAGGCTACTGAATTGGCTTTTGAATAT |
| SlLYK6-VIGS-R | GGGGACCACTTTGTACAAGAAAGCTGGGTCCACTGGAATTATAACCTCCT |
| SlLYK7-VIGS-F | GGGGACAAGTTTGTACAAAAAAGCAGGCTACAAGCTCCGAAACAAGGACAA |
| SlLYK7-VIGS-R | GGGGACCACTTTGTACAAGAAAGCTGGGTCTCCCTTGTCTTCTGTTCCCA |
| SlLYK4pro-F | GGGGACAAGTTTGTACAAAAAAGCAGGCTACAAATCTGCAGCCAAGAATTTTCC |
| SlLYK4pro-R | GGGGACCACTTTGTACAAGAAAGCTGGGTCTTTCAGTTCATTAGAATCAAG |
| SlEF-qRT-F | GGAACTTGAGAAGGAGCCTAAG |
| SlEF-qRT -R | CAACACCAACAGCAACAGTCT |
| SlACTIN7-qRT-F | CCTCAGCACATTCCAGCAG |
| SlACTIN7-qRT -R | CCACCAAACTTCTCCATCCC |
| SlLYK4-qRT-F | CCATGGTCAGATTGATCGATAA |
| SlLYK4-qRT-R | GGGCAATCTATGTGGTGACACT |
| SlLYK1-qRT-F | CCAGTTAACTCCCAATGTAAC |
| SlLYK1-qRT-R | CTGCTATACTGGTGGAGAACA |
| SlLYK6-qRT-F | AACGTTGGCGATGACATCCT |
| SlLYK6-qRT-R | TGATGGTTCAGAAGGCAGGG |
| SlLYK7-qRT-F | AGTTAAGCACCCTCTTGG |
| SlLYK7-qRT-R | CACTGTTGGGACCTGTCT |
| SlLYK12-qRT-F | TGAGGTCCATTGTTGTAG |
| SlLYK12-qRT-R | GTTGAAAATGCTTGAGTG |
| SlLYK13-qRT-F | AAATCAGTAGTGGTGGCT |
| SlLYK13-qRT-R | CTATTACTTTGAGGAGGC |
| SlWRKY33-qRT-F | GCATTACTGTCAACCATCGC |
| SlWRKY33-qRT-R | AACTTCGCGGATTCTCACTT |
| SlWRKY53-qRT-F | CCACAACCAACATCGCCAGAGAA |
| SlWRKY53-qRT-R | ACGGTGAATAGCCGCTACCTATCA |
| Bc3-F | GCTGTAATTTCAATGTGCAGAATCC |
| Bc3-R | GGAGCAACAATTAATCGCATTTC |

**Table S2** Vectors used in this study

| **Vectors** | **Description** | **Purpose** | **Source or reference** |
|---|---|---|---|
| pGWB5 | C-terminal GFP tag | Generation of *SlLYK4-GFP* overexpression lines; *SlCERK1*$^{K355E}$*-GFP*, *SlLYK4-GFP* and *SlLYK7-GFP* transient expression in *N. benthamiana* | |
| pGWB3 | C-terminal GUS tag | Generation of *SlLYK4 promoter-GUS* transgenic lines | Nakagawa et al. [1] |
| pGWB14 | C-terminal 3 × HA tag | *SlLYK4-HA* transient expression in *N. benthamiana* for CoIP | |
| pGWB17 | C-terminal 4 × Myc tag | *SlCERK1*$^{K355E}$*-Myc* transient expression in *N. benthamiana* for CoIP | |
| pUC19-GW-Myc | C-terminal 4 × Myc tag | *SlCERK1-Myc* transient expression in Arabidopsis protoplasts for CoIP | Laboratory stock |
| pUC19-GW-HA | C-terminal 3 × HA tag | SlLYK4-HA transient expression in Arabidopsis protoplasts for CoIP | |
| pAMPAT-YFPn/YFPc | C-terminal YFPn/YFPc tag | *SlCERK1*$^{K355E}$*-YFPn* and *SlLYK4-YFPc* transient expression in Arabidopsis protoplasts for BiFC | Chen et al. [2] |
| pTRV2 | TRV-based VIGS vector | Putative SlLYK4 silencing in tomato by VIGS | Liu et al. [3] |
| pDEST15 | N-terminal GST tag | Prokaryotic expression of the cytosolic kinase domains of SlLYK4 and SlCERK1 | Laboratory stock |

**Table S3** Accession numbers of genes mentioned in this study.

| Name | ID | Other name, remark[4] |
|---|---|---|
| *SlCERK1* | *Solyc07g049180* | *Bti9*, *LYK1*[5] |
| *SlLYK2* | *Solyc02g094010* | |
| *SlLYK3* | *Solyc03g121050* | |
| *SlLYK4* | *Solyc02g089900* | Tandem duplication with *SlLYK7*[4] |
| *SlLYK6* | *Solyc12g089020* | Kinase partly truncated |
| *SlLYK7* | *Solyc02g089920* | Tandem duplication with *SlLYK4* |
| *SlLYK8* | *Solyc09g083200* | Kinase truncated<br>Tandem duplication with *SlLYK9* |
| *SlLYK9* | *Solyc09g083210* | Tandem duplication with *SlLYK8* |
| *SlLYK10* | *Solyc02g065520* | |
| *SlLYK11* | *Solyc02g081040* | Tandem duplication with *SlLYK12* |
| *SlLYK12* | *Solyc02g081050* | Tandem duplication with *SlLYK11* |
| *SlLYK13* | *Solyc01g098410* | |
| *SlLYK14* | *Solyc06g069610* | |

| | |
|---|---|
| *SlLYK15* | *Solyc11g069630* |
| *AtCERK1* | *At3g21630* |
| *AtLYK2* | *At3g01840* |
| *AtLYK3* | *At1g51940* |
| *AtLYK4* | *At2g23770* |
| *AtLYK5* | *At2g33580* |
| *SlWRKY33* | *Solyc09g014990* |
| *SlWRKY53* | *Solyc08g008280* |
| *SlACTIN7* | *Solyc03g078400* |
| *SlEF*$\alpha$ | *Solyc06g005060* |

The accession numbers are found from the Sol genomics network for tomato and TAIR databases for Arabidopsis.


1. Nakagawa, T. *et al.* Development of series of gateway binary vectors, pGWBs, for realizing efficient construction of fusion genes for plant transformation. *Journal of Bioscience and Bioengineering* **104**, 34-41 http://dx.doi.org/10.1263/jbb.104.34 (2007).
2. Chen, D.Q. *et al.* S-acylation of P2K1 mediates extracellular ATP-induced immune signaling in *Arabidopsis*. *Nature Communications* **12**, 2750 2750 http://dx.doi.org/10.1038/s41467-021-22854-1 (2021).
3. Liu, Y.L., Schiff, M. & Dinesh-Kumar, S.P. Virus-induced gene silencing in tomato. *Plant Journal* **31**, 777-786 http://dx.doi.org/10.1046/j.1365-313X.2002.01394.x (2002).
4. Buendia, L., Wang, T., Girardin, A. & Lefebvre, B. The LysM receptor‐like kinase SlLYK10 regulates the arbuscular mycorrhizal symbiosis in tomato. *New Phytologist* **210**, 184-195 http://dx.doi.org/10.1111/nph.13753 (2016).
5. Zeng, L., Velásquez, A.C., Munkvold, K.R., Zhang, J. & Martin, G.B. A tomato LysM receptor-like kinase promotes immunity and its kinase activity is inhibited by AvrPtoB. *The Plant Journal* **69**, 92-103 http://dx.doi.org/10.1111/j.1365-313x.2011.04773.x (2012).
